# Fournier’s Gangrene and Intravenous Drug Abuse: an Unusual Case Report and Review of The Literature

**DOI:** 10.1515/med-2019-0114

**Published:** 2019-12-24

**Authors:** Michele Del Zingaro, Andrea Boni, Jacopo Adolfo Rossi De Vermandois, Alessio Paladini, Emanuele Lepri, Pietro Ursi, Roberto Cirocchi, Morena Turco, Gianluca Gaudio, Andrea Nogara, Ettore Mearini

**Affiliations:** 1 Department of Surgical and Biomedical Sciences, University of Perugia, Perugia, Italy; 2 Department of General Surgery and Surgical Specialties “Paride Stefanini”;, Sapienza University of Rome, Rome, Italy; 3 Division of General Surgery, Department of Surgical and Biochemical Sciences, University of Perugia, Perugia, Italy

**Keywords:** Fournier's gangrene, Necrotizing Fasciitis, Surgery, Infection

## Abstract

Fournier’s gangrene is a potentially fatal emergency condition characterized by necrotizing fasciitis and supported by an infection of the external genital, perineal and perianal region, with a rapid and progressive spread from subcutaneous fat tissue to fascial planes.

In this case report, a 52-year-old man, with a history of hepatitis C-virus (HCV)-related chronic liver disease and cocaine use disorder for which he was receiving methadone maintenance therapy, was admitted to the Emergency Department with necrotic tissue involving the external genitalia.

Fournier’s gangrene is usually due to compromised host immunity, without a precise cause of bacterial infection; here it is linked to a loco-regional intravenous injection of cocaine. A multimodal approach, including a wide surgical debridement and a postponed skin graft, was needed. Here we report this case, with a narrative review of the literature.

## 1 Introduction

Fournier’s gangrene was described for the first time in 1764 by Baurienne as an idiopathic, necrotizing lethal process in a man affected by gangrene of the genitalia. However, the origin of this clinical condition must be linked to Jean Alfred Fournier who described a series of fatal cases of idiopathic gangrene of the genitalia with a sudden onset in 5 young men in 1883 [1]. Fournier’s gangrene is a potentially fatal condition; it is characterized by necrotizing fasciitis and supported by an infection of the external genital, perineal and perianal region, with a rapid and progressive spread from subcutaneous fat tissue to fascial planes [2].

This emergency condition always requires a multimodal approach: antibiotic therapy, surgery followed by intensive care, and oxygen hyperbaric therapy [3].

Because of its rarity, most of the limited knowledge about Fournier’s gangrene derives from case reports and retrospective studies with small sample size [4].

Here, in order to improve the knowledge concerning Fournier’s gangrene, we describe an unusual case due to injection of cocaine into the superficial dorsal vein of the penis, followed by a comprehensive literature review.

## 2 Material and methods

We performed a narrative review of the literature by searching “*Fournier’s gangrene*”, “*necrotizing fasciitis*” on PubMed and Scopus (Table). Case reports, case series, and reviews were chosen and used to extract data regarding gender, age, comorbidity, pathogens, number of surgical debridements performed, peri-operative outcomes, intra-and post-operative complications, length of hospital stay, and number of hospitalizations in intensive care units. Two authors (AB, AP) independently performed online bibliographic searches to identify titles and abstracts of interest. Full texts of relevant articles were further assessed for inclusion in this study.

**Table 1:** review of the Literature up-to-date to July 2019

Reference	Year	Gender	N.of cases	Mean age	Surgical debridement	Days of hospital stay	Sepsi / ICU	Hyperbaric oxygen therapy	Pathogen	N.of deaths
Del Zingaro et al. [36]	2019	M	**1**	**76**	**1**	**ND**	**0**	**0**	Pseudomonas putida, Stenotrophomonas maltophilia, Staphylococcus haemolyticus and Staphylococcus Warneri	0
Arora et al. [37]	2019	50M	**50**	**53**	**1**	12.6	**ND**	**ND**	Escherichia coli, Staphylococci, Pseudomonas, Bacteroides, Streptococci	12
**Ali et al**. [38]	2019	M	**1**	**45**	**1**	**ND**	**1**	**0**	ND	0
Mostaghim et al. [39]	2019	M	**1**	**38**	**1**	**ND**	**0**	**0**	Escherichia coli, Enterococcus faecalis, Bacteroides thetaiotaomicron, Streptococcus agalactiae, Clostridium clostridioforme	0
Zhou et al. [40]	2019	M	**1**	**58**	**1**	**ND**	**1**	**0**	ND	0
Asian et al. [41]	2019	M	**1**	**12**	**0**	**8h**	**2**	**0**	Pseudomonas aeruginosa	1
Heijkoop et al. [42]	2019	ND	**14**	**ND**	**6**	**36**	9	**0**	ND	1
Paoneet al. [43]	2019	M	**1**	**72**	**>1**	**ND**	**2**	**0**	ND	0
Lin et al. [44]	2019	118M	118	**58**	**1**	**ND**	**ND**	**ND**	ND	17
Akella et al. [45]	2019	M	**1**	**37**	**1**	**ND**	**ND**	**0**	Staphylococcus aureus, Streptococcus, Anaerobic bacteroides	0
Klement et al. [46]	2019	M	**1**	**53**	**1**	**ND**	**1**	**ND**	ND	1
Onder et al. [47]	2019	M	**1**	**64**	**2**	**ND**	**ND**	**ND**	ND	0
Bersoff-Matcha et al. [48]	2019	39M 16F	**55**	**ND**	**1**	**ND**	**9**	**ND**	ND	3
Louro et al. [49]	2019	14M 1F	**15**	66.9	**3.3**	46.8	**ND**	**ND**	Escherichia coli, MRSA, Streptococcus pyogenes, Enterococcus faecium, Enterococcus cloacae, Klebsiella pneumoniae, Streptococcus epidermidis, Bacteroides fragilis, Corynebacterium, Candida albicans, Aspergillus fumigatus	0
Ünverdi et al. [50]	2019	13M	**13**	54.3	**1**	**42**	ND	**ND**	Bacteroides Fragilis, Escherichia coli, Klebsiella spp, Pseudomo-nas aeruginosa	**0**
Hong-Cheng et al. [51]	2019	56M 4F	**60**	**53**	1.17	**ND**	1	**0**	Escherichia coli, Enterococcus faecalis, Proteus mirabilis, Klebsiella pneumoniae, Peptostreptococcus, Pseudomonas aeruginosa	**1**
Rachana et al. [52]	2019	M	**1**	**50**	**1**	**18**	0	**0**	Fusobacterium varium, Escherichia coli, Bacteroides fragilis	**0**
Joury et al. [53]	2019	M	**1**	**51**	**1**	**ND**	ND	**0**	MRSA, Edwardsiella tarda, Klebsiella oxytoca, Prevotella	**0**
Selvi et al. [54]	2019	30M	**30**	62.9	**6**	**20**	9	**ND**	ND	**3**
Majdoub et al. [55]	2018	F	**1**	**70**	**-**	**-**	-	**-**	Escherichia coli, Bacteroides	**1**
Hahn et al. [56]	2018	33M 11F	**44**	54.4	**3.3**	**47**	18	**ND**	Polymicrobial flora (Escherichia coli, Entero- coccus, Staphylococcus, Klebsiella) (7), Monomicrobial flora (Staphylococcus, Escherichia coli, Klebsiella, Enterococcus, Candida (22)	9
Overholt et al. [57]	2018	M	**1**	**44**	**2**	**13**	0	**0**	Escherichia coli, Enterococcus avium, Gemella morbillorum	**0**
**Pehlivanli et al**. [58]	2018	19M 4F	**23**	65.9	**6**	**18**	ND	**ND**	Escherichia coli, Klebsiella, Staphylococci, Enterobacter	**5**
Kranz et al. [4]	2018	15 4M	154	62.7	**4.2**	26.6	104	**13**	Mixed flora (73), Streptococci (12), Staphylo- cocci (10), Enterococcus (10), Citrobacter (1), Pseudomonas (1), Candida (2)	**17**
Kobayashi et al. [59]	2018	M	**1**	**68**	**1**	**59**	1	**0**	Escherichia coli	**0**
Pandey et al. [60]	2018	M	**1**	**65**	**1**	**ND**	ND	**ND**	ND	**ND**
Matsuura et al. [61]	2018	M	**1**	**88**	**ND**	**ND**	ND	**0**	ND	**1**
Sen et al. [62]	2018	M	**1**	**47**	**1**	**18**	0	**0**	Rhizobium radiobacter	**0**
Elsaket et al. [63]	2018	43M 1F	**44**	**51**	1.33	26	6	**ND**	Staphylococcus aureus, Acinetobacter, Strep- tococcus pyogenes, Proteus mirabilis,	**5**
Heijkoop et al. [64]	2018	ND	**14**	**ND**	**6**	**36**	8	**0**	ND	**1**
Takano et al. [65]	2018	F	**1**	**44**	**1**	**ND**	ND	**0**	Streptococcus constellatus, Clostridium ramosum	**1**
Semenič et al. [66]	2018	**M**	**1**	30	2	16	**1**	**0**	Escherichia coli, Bacteroides fragilis, Prevo- tella oralis, Streptococcus anginosus	0
Abbas-Shereef et al. [67]	2018	**M**	**1**	71	>1	30	**1**	**0**	Pseudomonas aeruginosa, Klebsiella pneu- moniae, Candida albicans, Staphylococci, Group A Streptococcus	0
Wetterauer et al. [68]	2018	**20M**	**20**	66	4	ND	**15**	**0**	Escherichia coli, Klebsiella, Pseudomonas aeruginosa	3
Demir et al. [69]	2018	**49M** 25F	**74**	57.6	1.87	23.18	**ND**	**ND**	Escherichia coli, Staphylococcus aureus, Streptococci, Enterobacter, Pseudomonas aeruginosa, Bacteroides, Proteus, Clostrid-ium	6
Chen et al. [70]	2018	**M**	**1**	29	2	11	**1**	**0**	Streptococcus Agalactiae, Staphylococcus haemolyticus, Escherichia coli, peptostrepto-cocci, Prevotella corporis	0
Yuan et al. [71]	2018	**M**	**1**	62	1	ND	**1**	**ND**	Enterococcus avium, Escherichia coli	ND
Katsimantas et al. [72]	2018	**M**	**1**	68	2	17	**0**	**0**	Enterococcus faecalis, Streptococcus gordo- nii, Prevotella melaninogenica	0
Althunayyan et al. [73]	2018	**F**	**1**	36	2	31	**1**	**0**	Escherichia coli, Acinetobacter baumannii	0
Pittaka et al. [74]	2018	**F**	**1**	24	>1	14	**ND**	**ND**	ND	0
Taylor et al. [75]	2018	**F**	**1**	58	1	ND	**1**	**ND**	Bacteroides fragilis, Clostridium ramosum, Gram positive cocci	1
Dos Santos et al. [76]	2018	**29M 11F**	**40**	51.7	1.8	19.6	**9**	**ND**	ND	9
Fukui et al. *[77]*	2018	**M**	**1**	85	1	104	**1**	**0**	Streptococcus dysgalactiae, Escherichia coli, Staphylococci	0
Kuzaka et al. [78]	2018	**13M**	**13**	59.6	>1	31.9	**0**	**ND**	Enterobacteriaceae, Bacteroides, Parabacte- roides, Klebsiella, Staphylococcus, Lactoba-cillus acidophilus, Escherichia coli	0
Goel et al. [79]	2018	**M**	**1**	60	1	14	**0**	**0**	ND	0
Ghodoussipour et al. [80]	2018	**54M**	**54**	49.3	3.9	37.5	**53**	**ND**	ND	3
Tenόrio et al. [81]	2018	**99 M**, 25F	124	50.8	ND	21.7	**ND**	**1**	Escherichia coli, Proteus, Klebsiella, Pseu- domonas, Staphylococci, Enterococcus, Clostridium	32
Weimer et al. [82]	2017	**M**	**1**	55	>1	90	**1**	**0**	Parabacteroides distasonis, Prevotella melaninogenica, Fusobacterium nucleatum, Bacteroides	0
Wähmann et al. [83]	2017	**F**	**1**	46	3	ND	**1**	**ND**	Streptococci, Enterobacteria, gram+	0
Wangetal. [84]	2017	**M**	**1**	61	1	ND	**ND**	**ND**	Klebsiella pneumoniae	0
Yücel et al. [85]	2017	**UM, 14F**	**25**	54.3	2.4	21.4	**ND**	**0**	ND	1
ϋreyen et al. [86]	2017	18M, 11F	29	51.5	1.8	11.5	17	ND	Escherichia coli, Acinetobacter, Streptococci, Staphylococcus aureus, Pseudomonas, Klebsiella,	6
Dell'Atti et al. [87]	2017	M	1	**75**	1	28	1	0	ND	0
Yanaral et al. [88]	2017	54M	54	58.3	1.4	15.3	ND	0	ND	4
**Chia** et al. [89]	2017	42M, 17F	59	**56**	>1	19	11	ND	Streptococci, Escherichia coli, Prevotella	9
Kordahi et al. [90]	2017	M	1	**57**	>1	ND	ND	ND	ND	ND
Hong et al. [91]	2017	18M, 2F	20	61.8	1.55	36.9	15	0	Escherichia coli, Streptococci, Proteus, Kleb- siella pneumoniae, Enterococcus faecium, Pseudomonas aeruginosa, Staphylococcus aureus	5
Sanders et al. [92]	2017	M	1	**70**	2	ND	1	0	Escherichia coli, P. mirabilis	0
Ferretti et al. [93]	2017	19M, 1F	20	**56**	4	31.7	17	4	ND	3
Kumar et al. [94]	2017	M	1	**41**	2	15	1	0	Streptococcus anginosus, anaerobes, Gram -	0
loannidis et al. [95]	2017	20M, 4F	24	58.9	1	16	18	3	Escherichia coli (11), Klebsiella pneumoniae (3), Pseudomonas aeruginosa (3), Acineto-bacter baumannii (2), Proteus mirabilis (2), Providencia stuartii (1)	5
Bocchiotti et al. [96]	2017	M	1	**40**	3	ND	0	0	Escherichia coli, Streptococcus pyogenes, Prevotella loescheii	0
Choi et al. [97]	2017	F	1	**31**	1	17	0	0	Streptococcus anginosus, Pseudomonas, Clostridium	0
Sawayama et al. [98]	2017	M	1	66	1	ND	0	0	ND	0
Laureman et al. [99]	2017	125M, 43F	168	**ND**	>1	ND	92	0	Enterococcus faecalis, Klebsiella pneumo- niae, Escherichia coli, Clostridium difficile	6
Smith et al. [100]	2017	M	1	**50**	>1	ND	1	0	ND	0
Baek et al. [101]	2017	F	1	**57**	1	ND	1	ND	ND	0
Huang [102]	2017	M	1	**46**	1	ND	1	0	ND	0
Morais et al. [103]	2017	12M, 3F	15	**70**	ND	32	ND	0	Escherichia coli, Proteus, Staphylococcus aureus, Enterococcus faecalis	4
Okumura et al. [104]	2017	M	1	**70**	1	39	1	0	Klebsiella pneumoniae, Group G Streptococ- cus	0
Osbun et al. [105]	2017	ND	165	53.4	1.97	16.6	43	ND	ND	11
Kahn et al. [106]	2017	M	147	**52**	2.5	19	112	ND	ND	11
Misiakos et al. [107]	2017	47M, 15F	62	63.7	4.8	19.7	32	0	ND	11
Obi [108]	2017	4M		34.3	1	17.3	0	0	Staphylococcus aureus, Escherichia coli, Pseudomonas aeruginosa, Proteus mirabilis	0
Pernetti et al. [109]	2016	**M**	**1**	**70**	**1**	**21**	**1**	**ND**	ND	0
Faria et al. [110]	2016	**M**	**1**	**46**	**1**	**4**	**1**	**0**	ND	0
Ozkan et al. [111]	2016	**7M, 5F**	**12**	62.4	**5.7**	19.6	**ND**	**0**	Polymicrobial flora (6), monomicrobica (6)	0
Yoshino et al. [112]	2016	**M**	**1**	**64**	**1**	**33**	**1**	**0**	Streptococcus. alpha-emolitico	0
Crowell et al. [113]	2016	**M**	**1**	**54**	**3**	**18**	**1**	**0**	Rhizopus (zygomycosis)	1
Taken et al. [114]	2016	57M, **8F**	**65**	52.5	**2.5**	**9.2**	**13**	**0**	Escherichia coli, Streptococcus, Staphylococ- cus aureus, Enterobacter, Bacteroides, Pseu-domonas aeruginosa, Proteus, Clostridium	6
Wanis et al. [115]	2016	**M**	**1**	**28**	**1**	**14**	**1**	**0**	ND	0
Sheeny et al. [116]	2016	**M**	**1**	**48**	**2**	**ND**	**1**	**0**	Polymicrobial flora	0
Sarkut et al. [117]	2016	32M, 32F	**64**	**57**	**3**	16.6	**ND**	**ND**	ND	18
Sinha et al. [118]	2015	**F**	**1**	**30**	**1**	**ND**	**1**			0
Marín etal. [119]	2015	53M, **6F**	**59**	**68**	**ND**	24.4	**50**	**ND**	ND	15
Chalya et al. [120]	2015	82M, **2F**	**84**	**34**	**ND**	**28**	**ND**	**ND**		24
Namkoong et al. [121]	2015	**M**	**1**	**61**	**1**	**ND**	**1**	**0**	ND	0
Schulz et al. [122]	2015	**M**	**1**	**59**	**>1**	**ND**	**1**	**0**	ND	0
McCormack et al. [123]	2015	25M	**25**	56.6	**1.4**	**ND**	**3**	**ND**	Polymicrobial flora	5
Tarchouli et al. [124]	2015	64M, **8F**	72	**51**	**3.2**	28.7	**17**	**56**	Polymicrobial flora (37), Monomicrobial flora (1)	12
Paonam et al. [125]	2015	**M**	**1**	**65**	**1**	**ND**	**1**	**0**	Escherichia coli, Enterococcus	0
Oguz et al. [126]	2015	34M, **9F**	**43**	**52**	**>1**	**ND**	**43**	**0**	Polymicrobial flora (Escherichia coli 48%)	6
Asahata et al. [127]	2015	**M**	**1**	**70**	**1**	**ND**	**0**	**0**	Listeria monocytogenes, Escherichia coli	0
Ye et al. [128]	2015	**M**	**1**	**47**	**1**	**21**	**0**	**0**	Pseudomonas aeruginosa	0
Danesh et al. [129]	2015	**8M**	**8**	**44**	**>1**	**ND**	**ND**	**0**	Enterococcus, Pseudomonas, Staphylococcus haemolyticus, Proteus, Clostridium	3
Ossibi et al. [130]	2015	**M**	**1**	**60**	**1**	**ND**	**0**	**0**	ND	0
Mahmoudi et al. [131]	2015	**M**	**1**	**44**	**1**	**ND**	**1**	**0**	ND	0
Cochetti et al. [22]	2015	**2M**	**2**	42.5	**0.5**	**ND**	**2**	**1**	Staphylococcus warneri	1
Sarmah et al. [132]	2015	**M**	**1**	**68**	**1**	**1**	**1**	**0**	Bacteroides fragilis	1
Papadimitriou et al. [133]	2015	**M**	**1**	**56**	**1**	**90**	**1**	**0**	Polymicrobial flora	0
Ozsaker et al. [134]	2015	**M**	**1**	**69**	**1**	**ND**	**0**	**0**	ND	0
Garcia Marin et al. [135]	2015	53M, **6F**	**59**	**68**	**ND**	**ND**	**18**	**0**	ND	15
Toh et al. [136]	2014	**M**	**1**	**61**	**6**	**ND**	**1**	**0**	Polymicrobial flora	0
Parry et al. [137]	2014	**M**	**1**	**48**	**1**	**ND**	**0**	**0**	ND	0
Tena et al. [138]	2014	**M**	**1**	**73**	**1**	**55**	**1**	**0**	Actinomyces funkei, Clostridium hathewayi, Fusobacterium necrophorum	0
Matilsky et al. [139]	2014	**M**	**1**	**51**	**4**	**30**	**1**	**0**	Polymicrobial flora	0
Lee et al. [29]	2014	**3M**	**3**	50.7	**ND**	**ND**	**ND**	**ND**	ND	ND
Di Serafino et al. [140]	2014	**M**	**1**	63	1	ND	ND	ND	ND	0
Galukande et al. [141]	2014	**2M**	**2**	35.5	2.5	ND	0	0	ND	0
Tattersall et al. [142]	2014	**M**	**1**	61	2	47	1	ND	Escherichia coli	0
Omisanjo et al. [143]	2014	**11M**	**11**	51.9	>1	22.7	7	0	Klebsiella (10), Escherichia coli, Pseudomo- nas aeruginosa, no microbes (1)	0
Rubegni et al. [144]	2014	**2M**	**2**	58.5	1	ND	0.5	0	ND	1
Dinc et al. [145]	2014	**M**	**1**	51	>1	16	0	0	ND	0
Dayan et al. [146]	2014	**M**	**1**	27	>1	ND	0	0	ND	0
Ludolph et al. [147]	2014	**3M**	**3**	48.7	>1	ND	0	0	ND	0
Ozkan et al. [148]	2014	**7M, 5 F**	**12**	62.4	5.7	19.6	ND	0	Pseudomonas, Acinetobacter, Escherichia coli, Enterococcus, Stafilococcus aureus, Proteus, Corynebacterium, Polymicrobial flora (6)	ND
Shimizu et al. [149]	2014	**M**	**1**	74	2	ND	0	0	Proteus vulgaris, Prevotella denticola, Pepto- streptococcus species	ND
Ho et al. [150]	2014	**F**	**1**	78	1	14	0	0	ND	1
Aslanidis et al. [151]	2014	**F**	**1**	23	>1	ND	1	0	Candida albicans, Staphylococcus epider- midis, Klebsiella pneumoniae	0
D'Arena et al. [152]	2014	**M**	**1**	66	1	ND	0	0	ND	0
Perkins et al. [153]	2014	**M**	**1**	73	1	ND	0	0	Candida albicans	0
Sliwinski et al. [154]	2014	**M**	**1**	24	>1	ND	1	0	ND	0
Agostini et al. [155]	2014	**M**	**1**	64	2	58	1	1	Staphylococcus epidermidis, Proteus mirabi- lis, Enterococcus faecalis	0
Oymaci et al. [156]	2014	10M, **6F**	**16**	61.2	4.44	25.5	ND	0	Escherichia coli, Acinetobacter baumannii, Proteus mirabilis, Staphylococcus aureus, Enterococcus	3
Eskitascioglu et al. [157]	2014	76M, **4F**	**80**	53.5	1.55	34.78	ND	0	Polymicrobial flora (14), Escherichia coli, Staphylococcus aureus, Enterococcus, Acine-tobacter baumanii, Staphylococcus epider-midis, Proteus, etc.	3
Yilmazlar et al. [158]	2014	81M, 39F	120	58	3	14.5	48	0	Escherichia coli, Streptococci, Enterococci, Staphylococci, Klebsiella, Pseudomonas, Proteus, fungi	25
Akbulut et al. [159]	2014	**M**	**1**	***77***	1	20	0	0	Escherichia coli	0
Coyne et al. [160]	2014	**M**	**1**	48	1	ND	0	0	ND	0
Li et al. [161]	2014	48M, **3 F**	**51**	49.7	>1	17	ND	0	Escherichia coli, Streptococcus, Staphylococcus aureus, Pseudomonas, Proteus, Clostrid-ium, Bacteroides	
Oyaert et al. [162]	2014	**M**	1	43	1	63	**1**	0	Atopobium	0
Lee et al. [163]	2013	**M**	1	47	>1	ND	**0**	0	Enterococcus, Enterobacter	0
Abate et al. [164]	2013	**M**	1	63	1	21	**0**	0	Enterococcus faecalis, Citrobacter freundii, Pseudomonas aeruginosa, Escherichia coli, Bacteroides fragilis, Bacteroides ovatus	0
Anantha et al. [165]	2013	**M**	1	59	1	16	**1**	0	Streptococcus anginosus	0
Benjelloun et al. [166]	2013	**44M, 6F**	50	48	2.5	21	**11**	0	Escherichia coli, Klebsiella	12
Pastore et al. [167]	2013	**M**	1	60	>1	34	**0**	1	Streptococcus A	0
Eray et al. [168]	2013	34M, 14F	48	53.7	ND	25.3	**ND**	0	ND	9
Bjurlin et al. [169]	2013	40M, 1F	41	49	ND	ND	**ND**	ND	Polymicrobial flora (34), Bacteroides (43.9%), Escherichia coli (36.6%), Prevotella, Streptococci, Staphylococcus aureus	2
Park et al. [170]	2013	**M**	1	59	>1	ND	**0**	0	ND	0
Subramaniam et al. [171]	2013	**M**	1	80	3	ND	**1**	0	Escherichia coli, Anaerobes	0
Sabzi Sarvestani et al. [172]	2013	28M	28	44.6	2.2	17.22	**ND**	0	Escherichia coli, Bacteroides, Streptococci, Enterococci, Staphylococci, Pseudomonas, Klebsiella, Proteus	10
Katib et al. [173]	2013	20M	20	55.95	1.7	22.3	**1**	0	Acinetobacter spp. (most common)	0
Czymek et al. [174]	2013	72M, 14F	86	57.9	4	52	**52**	ND	Polymicrobial flora (71), Escherichia coli, Enterococci, Streptococci, Pseudomonas, Staphylococci, etc.	14
Akilov et al. [175]	2013	28M	28	47.1	3.5	24.4	**8**	0	Monomicrobial flora (18), Staphylococci, Streptococci, Enterobacter, Pseudomonas	0
Bakari et al. [176]	2013	**10M**	10	50.5	ND	ND	**ND**	0	ND	ND
Avakoudjo et al. [177]	2013	**ND**	72	ND	ND	72	**ND**	ND	Escherichia coli, Staphylococci, Pseu- domonas aeruginosa, Klebsiella	7
Chan et al. [178]	2013	**M**	1	78	1	ND	**1**	0	Escherichia coli	0
Chan et al. [179]	2013	**M**	1	49	15	ND	**0**	0	Escherichia coli, Streptococci, Arcanobacte- rium	0
Aliyu et al. [180]	2013	43M	43	37.82	>1	28	**ND**	0	Polymicrobial flora (27)	6
Ozkan et al. [181]	2013	**F**	1	43	4	ND	**1**	0	ND	0
Khan et al. [33]	2013	**M**	1	47	3	ND	**1**	0	ND	0
Vyas et al. [182]	2013	30M	30	39.6	2.2	*9.7*	**ND**	0	Escherichia coli, anaerobes, Streptococci, Pseudomonas, Staphylococci	6

ICU=intensive care unit ND=not defined

## 3 Case report

**Figure 1:**
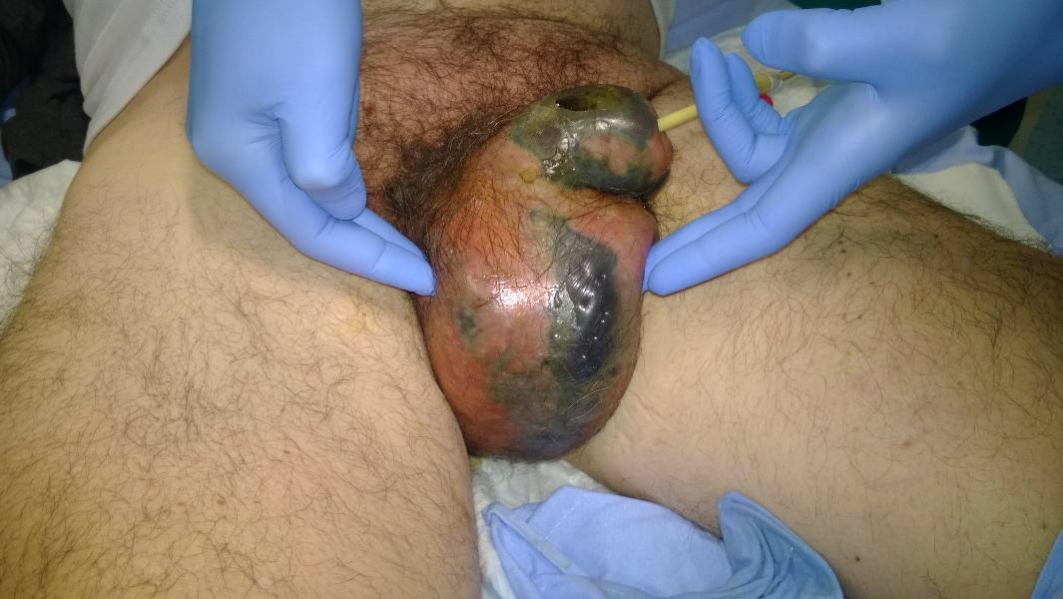
The physical examination was notable for necrotic-appearing tissue in the entire penis and scrotum, with areas of induration and crepitus

A 52-year-old man with a history of a cocaine use disorder, who was in methadone maintenance therapy and affected by HCV-related chronic liver disease, was admitted to the Emergency Department of a high-volume hospital. At admission to our institution, he presented with fever, acute renal impairment, anuria, poor hygienic conditions, and necrotic tissue involving the external genitalia (Figure 1). The laboratory tests showed 29 x 10^9^/L white blood cells with 95% neutrophils, haemoglobin 15.6 g/dl, glucose 103 mg/dl, aspartate transaminase 79 UI/L, alanine transaminase 68 UI/L, creatinine 2.58 mg/dl, C-reactive protein 56.2 mg/dl, procalcitonin >100 ng/ml. HIV testing was negative. The patient reported no other urological symptoms at hospital afdmission. He had a Charlson Comorbidity Index score of 2 and an Eastern Cooperative Oncology Group (ECOG) of 1, with no referring major comorbidities.

A scrotal ultrasound examination was performed. It showed the right testis augmented in volume with completely altered echogenicity, augmented vascular sign and hypoechoic areas. The left epididymis and involucres had irregular echogenicity. The left testis appeared to have irregular echogenicity and was hypervascularized with hypoechoic areas. A left hydrocele was present. Computed tomography (CT) was performed. It confirmed phlogosis and edema of the scrotum, with the right testis unrecognizable. Skin, subcutaneous planes, spermatic cord were thickened. Inguinal bilateral and right external iliac lymphadenopathy was described on CT.

The patient underwent resuscitation intravenous fluid support; antibiotic therapy was administered with tigecycline and meropenem. A single, prompt, extended surgical debridement of external genital, perineal, perianal and infrapubic regions to healthy tissue was performed. The patient also underwent at the same time right orchiectomy.

The microbiologic culture of the wound specimen revealed *Staphylococcus lugdunensis* with tigecycline susceptibility. Urine and blood samples cultures were negative. Tigecycline and meropenem were administered until discharge.

The anuric condition persisted for 24 hours; then polyuria developed, but with a renal impairment that required treatment with dialysis.

Five days after the surgical debridement the patient reported the injection of cocaine into the superficial dorsal vein of the penis.

The histologic report confirmed an inflammatory necrotizing process of subcutaneous tissue that expanded to skin, testicular and epididymis parenchyma, rete testis and peritesticular tissue.

No other wound treatments were performed for the wide extension of involved cutaneous area and the correct development of granulation tissue.

The patient was discharged 17 days after the surgical debridement and was admitted to the waiting list for a skin graft, which was successfully performed 1 month later.

**Ethical approval**: The research related to human use has been complied with all the relevant national regulations, institutional policies and in accordance the tenets of the Helsinki Declaration, and has been approved by the authors' institutional review board of Perugia University.

Written informed consent was obtained from the patient.

## 4 Discussion

Fournier’s gangrene is a surgical emergency characterized by necrotizing fasciitis of the genital, perineal and perianal soft tissue. It is a rare condition, representing 0.02% of hospitalizations, with an estimated incidence of 1.6 for 100.000 males/year [5]. This condition affects both males and females. Males are more affected than females with a ratio 10:1, and age of onset is becoming older (between 60s and 70s) [6].

The patient in our case of necrotizing fasciitis was 52 years old. Fournier’s gangrene was initially described as an idiopathic process, which has been found to be true in only a few cases. Often the initial cause is an infection involving the ano-rectum (30-50%), uro-genitalia (20-40%) and genital skin (20%). [7, 8, 9]. Infection results in inflammation and edema, which leads to obliterating endarteritis of the subcutaneous vessels [10]. The resulting lower blood support leads to peripheral dissection, with consequent spread of infection between the subcutaneous tissue and the skin. The reduction of the blood supply therefore generates gancrena [11].

This necrotizing fasciitis may be due to a condition of compromised host immunity, like diabetes, alcoholism, human immunodeficiency syndrome (HIV), lymphoproliferative diseases, arterial hypertension, renal and hepatic insufficiency, obesity, dementia, tobacco consumption, chronic steroid abuse, chemo- and radiotherapy, or cancer and surgical treatment [12, 13, 14, 15, 16, 17, 18, 19, 20]. In our case, a correlation between gangrene and a patient with a history of cocaine abuse undergoing methadone substitution treatment has been highlighted. The patient was also affected by HCV-related chronic liver disease.

The pathogen involved is both aerobic and anaerobic, gram-negative and gram-positive. Some authors suggest the use of three different antibiotic classes to start an empiric treatment to cover all types of pathogen. In most of the cases, a polymicrobial infection (54%) is demonstrated, and *Escherichia coli* is the most frequently isolated pathogen (46.6%). The pathogens with a lower incidence are the streptococcus, bacteroides, enterobacter, staphylococcus, enterococcus, pseudomonas, corynebacterium, and *Klebsiella pneumoniae* [21]. Our review confirms that *E. coli* is the most involved pathogen (53,1%) and a polymicrobial infection the most common cause (68%) of Fournier’s gangrene. However, many authors suggest the use of broad-spectrum penicillin or third-generation cephalosporins, an aminoglycoside (e.g. gentamicin) and metronidazole or clindamycin [11]. In our case tigecycline and meropenem were administered to cover aerobic gram-positive and gram-negative pathogens, as well as anaerobic gram-positive and gram-negative pathogens. The administration was related to renal-function impairment.

The risk of a fatal event makes this necrotizing fasciitis an emergency clinical condition. Prompt management is mandatory; hemodynamic support with resuscitation with fluids, board-spectrum parental antibiotics and surgical debridement of the involved region are the main procedures [22, 23]. Thanks to these approaches, the mortality linked to Fournier’s gangrene has dropped from between 20% and 88% to lower than 10% [24, 25]. On the basis of the data we collected, the reported mortality was 14,1 %. In our case, the patient survived the acute condition, and he is still alive.

The surgical debridement must be performed within a few hours of hospitalization, and the removal of necrotic tissue helps in stopping progression of necrotizing fasciitis and in reducing the risk of death [26]. Nevertheless, Proud et al, in a retrospective study of 219 patients found no differences in mortality in patients treated within 24 hours and those not treated. The authors linked this result to the severity of the infection [27]. For some authors (Chowla et al), more than one surgical debridement is necessary to obtain adequate infection control [28]. From our review of literature, more than one surgical debridement was performed in more than 60% of cases. In our case, we performed one surgical debridement, with the goal of obtaining a partial resection of viable tissue adjacent to the necrotic one.

Negative pressure wound therapy (NPWT) may represent a solution to the risk of infection of the large open wound that usually remains after a surgical debridement, since the patient’s poor condition it may be difficult to create a skin flap with which to cover the wound [29]. In NPWT the wound is exposed to a sub-atmospheric pressure between 50 and 125 mm Hg in order to increase blood supply, migration of inflammatory cells, and removal of exudates [11]. According to Chang et al, NPWT allows less frequent wound medication and reduction of pain and length of hospital stay [30].

The use of hyperbaric oxygen therapy (HBOT) is increasing in the management of Fournier’s gangrene, but evidence of efficacy is lacking [31]. In the HBOT treatment, the patient inhales 100% O2 in increased ambient pressure (2 – 3 atmospheres). HBOT has bactericide and bacteriostatic effects on anaerobic pathogens, in particular. It also improves bacterial lysis by leukocytes and stimulates collagen formation and superoxide dismutase with tissue survival [11,13]. Some authors have reported the range of mortality to be between 16% and 30%, whereas for the patients who undergo HBOT, the mortality is found to be approximately 17.6%.

In our case, our patient obtained a complete resolution of the necrotizing process without NPWT or HBOT, and a skin graft was then performed.

Cocaine, as described by Burnett [32], could be associated with priapism, and when administered into the corpora cavernosa, it could produce a prolonged erection [33]. In our knowledge, only two other cases of Fournier’s gangrene associated with penile injection of cocaine [33, 34] and three cases of penile necrosis [35] have been described. In both cases of Fournier’s gangrene, the necrosis was limited to the penis. The mechanism behind the necrotizing fasciitis after intra-corpora cavernosa injection of cocaine could be dual: cocaine has an intensive vasoconstrictive action that could lead to dermal necrosis that could be complicated by superinfection [33] or by inoculation of infective agents [34]. In our case, we believe that the inoculation of infective agents was the most plausible mechanism, since a skin commensal bacterium was involved.

## 5 Conclusion

Fournier’s gangrene is a potentially fatal condition that must be treated in a multimodal setting.

Here we report a rare case of genital and perineal necrotizing fasciitis after a loco-regional intravenous injection of cocaine.

To offer the patient the possibility of survival, a prompt application of a multimodal approach with intravenous fluid support, antibiotic therapy and aggressive surgical debridement is mandatory.
